# Biomechanics of Pulmonary Autograft as Living Tissue: A Systematic Review

**DOI:** 10.3390/bioengineering9090456

**Published:** 2022-09-08

**Authors:** Francesco Nappi, Sanjeet Singh Avtaar Singh

**Affiliations:** 1 Department of Cardiac Surgery, Centre Cardiologique Du Nord, 93200 Saint-Denis, France; 2 Department of Cardiothoracic Surgery, Aberdeen Royal Infirmary, Aberdeen AB25 2ZN, UK

**Keywords:** pulmonary autograft, living tissue, biomechanics ross operation, pulmonary autograft expansion, finite element analysis

## Abstract

Introduction: The choice of valve substitute for aortic valve surgery is tailored to the patient with specific indications and contraindications to consider. The use of an autologous pulmonary artery (PA) with a simultaneous homograft in the pulmonary position is called a Ross procedure. It permits somatic growth and the avoidance of lifelong anticoagulation. Concerns remain on the functionality of a pulmonary autograft in the aortic position when exposed to systemic pressure. Methods: A literature review was performed incorporating the following databases: Pub Med (1996 to present), Ovid Medline (1958 to present), and Ovid Embase (1982 to present), which was run on 1 January 2022 with the following targeted words: biomechanics of pulmonary autograft, biomechanics of Ross operation, aortic valve replacement and pulmonary autograph, aortic valve replacement and Ross procedure. To address the issues with heterogeneity, studies involving the pediatric cohort were also analyzed separately. The outcomes measured were early- and late-graft failure alongside mortality. Results: a total of 8468 patients were included based on 40 studies (7796 in pediatric cohort and young adult series and 672 in pediatric series). There was considerable experience accumulated by various institutions around the world. Late rates of biomechanical failure and mortality were low and comparable to the general population. The biomechanical properties of the PA were superior to other valve substitutes. Mathematical and finite element analysis studies have shown the potential stress-shielding effects of the PA root. Conclusion: The Ross procedure has excellent durability and longevity in clinical and biomechanical studies. The use of external reinforcements such as semi-resorbable scaffolds may further extend their longevity.

## 1. Introduction

### Pulmonary Autograft, a Biological Entity for a Modern Clinical Challenge

**Figure 1:**
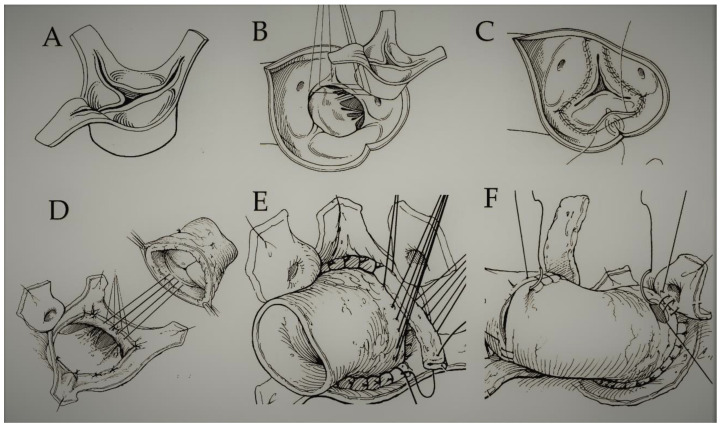
(**A**–**F**) The pulmonary autograft can be implanted using 2 methods. (**A**–**C**) Subcoronary implantation or (**D**–**F**) free-end/mini-root technique. In the subcoronary technique, the pulmonary valve is taken and inserted only with its leaflets and annulus. In the mini-root technique, the pulmonary valve is implanted with its pulmonary trunk so that the PA is withdrawn from the infundibulum of the right ventricle, respecting its morphology. Abbreviation; PA, pulmonary autograft.

**Figure 2:**
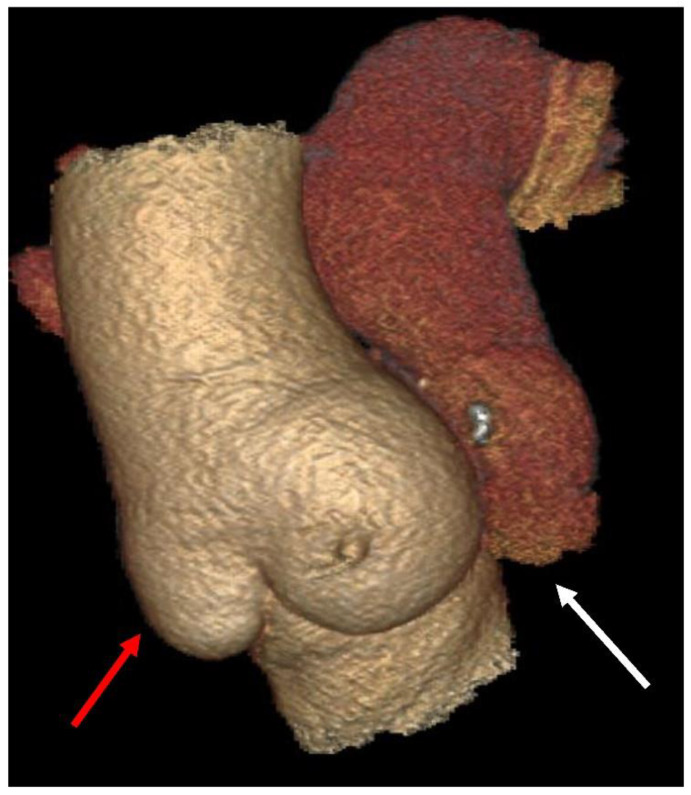
Ross operation after 23 years. Pulmonary autograft (**red arrow**), pulmonary homograft (**white arrow**).

A pulmonary autograft is used as a living tissue to replace a diseased aortic valve. This procedure, which involves the simultaneous insertion of a homograft in the pulmonary position, was first reported in humans by Donald Ross in 1967, following successes in animal model studies by the Stanford group. Figure 1 and Figure 2 demonstrate the different implantation techniques of a pulmonary autograft [1,2].

Aortic valve surgery accounts for approximately 85,000 procedures performed annually in the United States [3] for which guidelines and position papers from professional societies recommend Ross’s operation as a viable option for congenital and acquired left ventricular outflow tract disease in selected cases [4,5,6,7,8,9,10,11,12,13,14].

Biomechanical assessments associated with histology studies of the aortic valve and aortic root offer a substantial advantage in guiding the choice of the ideal valve substitute in patients requiring a replacement of the aortic valve. Since the choice of the ideal substitute must be carefully tailored to the individual patient, both biomechanics and histology, therefore, play a crucial role in influencing the long-term results [15,16,17,18,19,20,21,22,23,24,25,26,27,28,29,30]. The biomechanical features of a pulmonary autograft (PA) make it a suitable option among children and young adults as the category of patients who benefit most extensively from Ross procedures. Another significant special population is represented by women of childbearing age and patients with contraindications to oral anticoagulants [4,31,32,33,34]. The use of PA as living tissue has disclosed remarkable benefits related to the potential of the somatic growth of cardiovascular structures and the avoidance of anticoagulants whose lifelong administration is required with conventional mechanical prostheses [35,36,37]. However, the concerns related to the use of pulmonary autografts are due to the potential progressive expansion that is associated with persistent pulmonary valve leaflet integrity. The pathoanatomic phenomena of pulmonary autograft dilatation range from 20 to 40%, conditioning reoperation, which is not uncommon [38,39,40,41,42,43,44,45,46,47,48,49,50]. 

Several human studies and animal models have reported increased stresses on the pulmonary autograft root and leaflet compared to similar components of the native aorta, revealing the long-term durability of pulmonary autografts subjected to the regime of systemic pressure [23,24,25,26,30,51,52,53,54]. In particular, an animal model integrated with mathematical models led to the understanding of the mechanical stresses of the PA root and leaflet during the growth phase, thereby offering a substantial contribution to the knowledge of the durability of the PA over time and suggesting the regions more prone to dilation. The reported evidence is useful for achieving the best implant technique during the Ross procedure [23,24,25,26]. 

Two independent groups of investigators assumed a non-linear constitutive stress–strain relationship, as evidenced by mechanical tests, to examine the mechanical differences between the two vessels along the circumferential and longitudinal directions [23,24,25,26,29,51,52]. We generated a regular hexahedral mesh, in which each finite element was associated with eight nodes with three translational degrees of freedom, to measure the pulmonary autograft expansion [25,28,29]. Furthermore, the pulmonary autograft was reinforced with a semi-resorbable composite device that was 3D-ideally designed to prevent the degeneration and failure of the PA [23,26,55]. 

The relationship between the pathological process that occurs in the PA wall and the stress levels to which a pulmonary autograft is subjected to has been largely described [16,17,18,19,20,30]. Specifically, we worked to explain the mechanisms that modulate the structural integrity and flexibility of a pulmonary autograft, focusing on the pathophysiological processes leading to apoptosis and the proliferation of vascular smooth muscle cells under conditions of high-stress levels [21,24]. The final results of the regulatory remodeling pathways of the extracellular matrix within the PA reinforced with a semi-absorbable scaffold are described in the presence of high stress–strain conditions both in the valve leaflet and pulmonary root [26,28]. 

The aim of this systematic review is to examine observational and prospective reports on the biomechanical features of PAs, which may determine the subsequent dysfunctioning of the pulmonary autograft, thus causing an increase in mortality and morbidity. Moreover, we examined the biomechanics of pulmonary autografts in relation to the different techniques of implantation and in the presence of external reinforcement in adults and during the somatic growth phase. The histology of living pulmonary autograft tissue was studied to improve the understanding of the potential determinants of success using pulmonary autografts. 

We believe that the data presented herein can provide a further understanding of the PA mechanics after the Ross procedure and assist physician–patient discussions about the risks, benefits, and expectations after the use of pulmonary autografts to treat aortic valve disease. 

We present the following article in accordance with the PRISMA reporting checklist.

## 2. Methods

### 2.1. Search Strategy

This systematic review was planned drawing advice from the work of Stroup et al. [56]. One reviewer and the coordinating center were established in France and one reviewer in the UK to collect data [56,57,58,59]. 

The electronic search was driven by Pub Med (1996 to present), Ovid Medline (1958 to present), and Ovid Embase (1982 to present) and run-on 1 January 2022 with the following targeted words: biomechanics of pulmonary autograft, biomechanics of Ross operation, aortic valve replacement and pulmonary autograph, aortic valve replacement and Ross procedure, aortic valve replacement and Ross operation. The terms biomechanics of pulmonary autograft and biomechanics of Ross operation were coupled to pulmonary autograft dysfunction or failure. A large number of publications were assessed from 1979 and 2022 in patients who received a pulmonary autograft to treat aortic valve replacement at adult age and during somatic growth. To ensure fulfilment, a search of the Cochrane library (2010 to present) was conducted using the following keywords: autograft aortic valve replacement and biomechanics of autograft aortic valve replacement, or Ross procedure and Ross operation coupled with the biomechanics of Ross procedure or Ross operation to access titles and abstracts for detailed analysis of the manuscripts. This review followed the Preferred Reporting Items for Systematic Reviews and Meta-analyses (PRISMA) reporting guidelines [56,57,58,59]. 

### 2.2. Data Extraction 

Searches retrieved 2280 results. Two reviewers (FN, SSAS) screened the literature and analyzed the titles and abstracts, against the predefined inclusion and exclusion criteria, of all the selected studies judged pertinent to the systematic review. Case reports, conference presentations, editorials, expert opinions, and observational studies were excluded. A statistical reviewer (FN) assessed whether inclusion and exclusion were performed accurately. All disagreements were resolved by discussion between the investigators if they needed to reach an agreement.

We conferred particular attention to the reports that had a follow-up of >90% and investigations involving ≥30 patients, reflecting the high level of center experience. In cases where multiple publications emerged from the same patient population (see Deutsch registry for Ross operation), we selected the most recent report and meticulously analyzed the statistical methodology used. Randomized controlled trials were preferred where possible [56,57,58,59]. 

The following variables were extracted: study data and location, study type, study period, number of patients enrolled, mean age, mean length of follow-up, and major findings. The potential age heterogeneity of the patients included in the publications was overcome by considering 2 categories of recipients of the Ross procedure. (1) Consecutive series of Ross procedures performed in children were included; (2) consecutive series of Ross procedures performed on adult and child populations were included.

The target outcomes incorporated indications for surgery and the procedure used. Pulmonary autograft failure due to biomechanical alteration after aortic valve replacement was evaluated in patients who received autograft aortic valve or root replacement. Outcome events were discussed using the 2020 ACC/AHA, and 2021 ESC/European Association for Cardiothoracic Surgery guidelines [3,60]. 

The primary outcomes were early- and late-PA failure as absolute values or rates. Secondary endpoints included mortality [56,57,58,59]. 

## 3. Results

**Figure 3:**
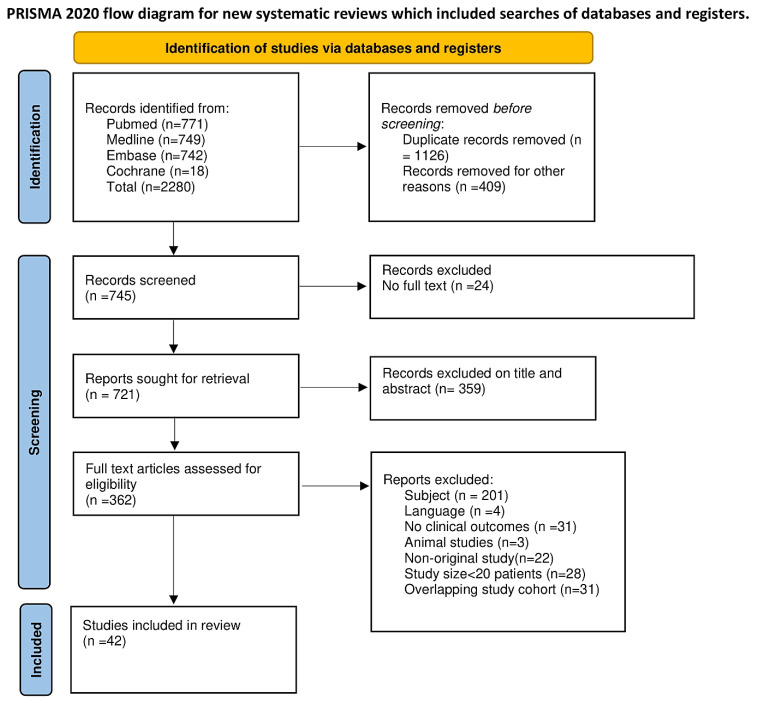
Prisma flow diagram.

A total of 362 studies were evaluated, of which 42 studies were included and 320 excluded in the final analysis due to them not meeting the eligibility criteria. The full PRISMA flow diagram outlining the study screening process is reported in Figure 3. 

The PRISMA 2020 Checklist items are enclosed in Table S1 in Supplementary Materials: Prisma checklist for Biomechanics of Pulmonary Autograft as Living Tissue: A Systematic Review. The details of the eligibility criteria of the manuscripts are reported in Table 1 and Table 2.

A total of 8468 patients were included (7796 in the pediatric cohort and young adult series and 672 in the pediatric series) and International Guidelines was reported [3,13,28,31,32,33,34,37,46,47,60,61,62,63,64,65,66,67,68,69,70,71,72,73,74,75,76,77,78,79,80,81,82,83,84,85,86,87,88,89,90,91]. 

**Figure 4:**
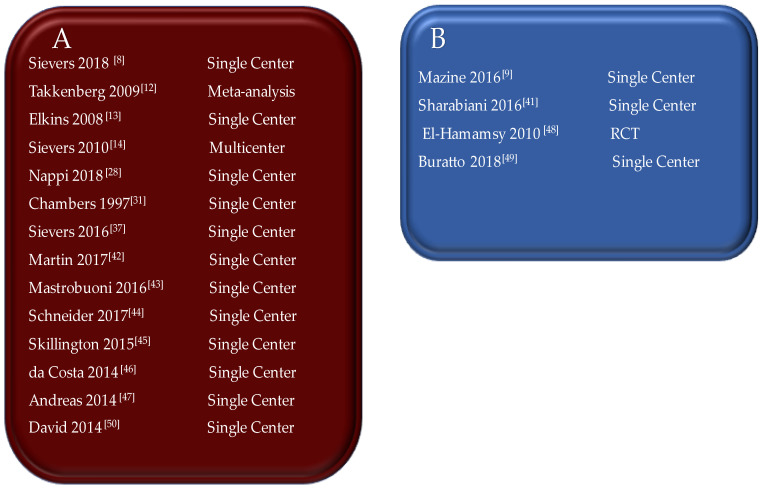
(**A**) Studies reporting long-term outcomes (>15 Years) of the Ross procedure in adult and pediatric populations; (**B**) Studies comparing the Ross procedure to homograft and conventional prosthesis; Abbreviation; RCT, randomized clinical trial. [8,9,13,14,28,31,37,41,42,43,44,45,46,47,48,49,50].

The main discovery of this study revealed the considerable experience with the use of the Ross procedure accumulated by various institutions around the world. Late rates of biomechanical failure and mortality were substantially low when similar populations were compared for age, such as youths and adults who received the use of a pulmonary autograft and matched with the healthy general population—Figure 4. [8,9,12,13,14,28,31,36,37,41,42,43,44,45,46,47,48,49,50]. 

Again, the evidence suggests that the survival benefit associated with the use of a pulmonary autograft was strongly related to the persistence of the biomechanical features of pulmonary autografts and this condition was widely disclosed in children and young adults. The results proved that the biomechanical features specific to PAs compared to other substitutes (mechanical or biological) were the primary factor related to long-term mortality after treating aortic valve disease in this age range [9,41,42,49,92]. In fact, the advantages of the avoidance of lifelong anticoagulant treatment, the better haemodynamic features of pulmonary autografts, and their increase in sizing that matches somatic growth were noteworthy. Living tissue (pulmonary autograft) undergoes favourable remodelling when translated into the aortic position as clearly demonstrated in several biomechanical studies [23,25,26,28,29,30,51,52]. 

### 3.1. Proposed Advantages of Pulmonary Autografts as Valve Substitutes: The Living Aortic Root

The aortic root works as a sophisticated frame that includes four crucial elements represented by the aortic annulus, the aortic leaflets, the sinuses of Valsalva, and the sinotubular junction. We learned that the aortic valve exercises a compliant morphofunctional role limited to opening and closing action dependent on the generated transvalvular pressure gradient. However, clinical and experimental evidence has suggested a much more complex role that is performed by each component of the aortic root, which therefore functions as a living dynamic structure. In the aortic root, all components work together to form a coordinated functional unit [15,16,17,18,19,20,21,22,23,24,25,26,27,28,29,30]. Dagum et al. studied the aortic root from a functional point of view, revealing that the aortic root (AR) is subjected to multiple complex three-dimensional deformations in every part of the cardiac cycle. In particular, the deformations involving the aortic annulus, the sinuses of Valsalva, and the sinotubular junction are expansive and contractile. They play a crucial role in decreasing the stress grade localized in the aortic leaflet, favoring the required functional laminar flow during systole to improve the coronary flow reserve in systole and diastole [93]. 

Studies targeting the microstructure of the aortic valve leaflets have disclosed the further intricacies and sophistication of the aortic root. Histological evaluations were reported in a pivotal study by El-Hamamsy et al., who demonstrated the presence of a monolayer of valve endothelial cells lining the ventricular and aortic sides of the cusps [17]. On the contrary, the extracellular matrix that forms the architecture of the cusp is made up of a mixed population of interstitial valve cells such as smooth muscle cells, fibroblasts, and myofibroblasts. The observations subsequently published by El-Hamamsy et al. are of substantial importance, focusing on the mechanism of mechanotransduction, in which valvular endothelial cells perceive and respond to changes induced by shear stress. In this way, valvular endothelial cells can translate mechanical stimuli into biological signals [18]. Likewise, the investigators suggested that endothelium-dependent signals can regulate the biomechanical ownership of aortic valve leaflets in response to their humoral habitat [18]. Again, the evidence provided in El-Hamamsy’s study demonstrated that interstitial valvular cells have both intrinsic secretory and contractile properties, thereby playing a crucial role in the generation, maintenance, and repair of the extracellular matrix, which is essentially structured from elastin, collagen, and glycosaminoglycans [16,17]. Subsequently, Warnock et al. revealed that vasoactive agents such as angiotensin II (Ang II) and 5-hydroxytryptamine (5-HT) had the potential of increasing the elastic modulus of aortic valve tissue in a time-dependent manner [94]. Finally, the innervation of the aortic valve leaflets supported by microscopic evaluation is fundamental because it has revealed a rich network of intrinsic nerves. The latter are supposed to exert significant action in the process of modulating the responses of the aortic valve to various haemodynamic conditions and humoral stimuli [16].

Once the above evidence has been translated into the surgical principles that guide the use of pulmonary autografts, it offers an understanding of how the complex architecture and aortic root function are crucial to the Ross operation. The patient’s diseased aortic valve is replaced by viable tissue that forms a living valve substitute. It preserves the structural and functional unity of the neoaortic root with favorable long-term clinical results. Therefore, given its characteristics, a pulmonary autograft can be considered the only substitute that potentially guarantees the long-term viability of the neoaortic valve. Conversely, if we consider all other aortic valve substitutes, they reveal the anatomofunctional features of nonliving valve substitutes. Although homografts, once removed under sterile conditions and not subjected to a cryopreservation process that induces a histological transformation, are kept in a tissue culture medium and implanted at the earliest available opportunity, they have no proven longevity and durability. In fact, although designed to favor long-term profitability, they have demonstrated a histological transformation towards acellularity after a few weeks following implantation [6,7,16,17,19,93,94,95]

### 3.2. Insights on Adaptative Remodelling of the Pulmonary Autograft

The biological process that promotes PA dilation is fully understood. A promising boost to our knowledge has been provided by molecular biology and proteomics.

A key determinant that makes pulmonary autografts suitable to withstanding systemic pressure stresses is their capacity for tissue remodeling [20,21,22,23,24,25,26,27]. This adaptive remodeling offers PAs the morphofunctional characteristics of an ideal substitute when they are transposed into the aortic position. Rabkin-Aikawa et al. revealed that this potential for intrinsic remodeling linked to the histological properties of PAs leads to the mimicking of the highly refined anatomy and function peculiar to the native aortic root. We know that the expressed remodeling is substantially dependent on valvular endothelial and interstitial cells, which are favored by genomic activation, leading to a change in phenotype. This process is essentially related to the exposure of the pulmonary valve to higher systemic pressure and the crucial step of this adaptative phenomenon is the expression of EphrinB2 by the endothelial cells of PAs. The increased level of EphrinB2 is a distinctive feature of the left side of the heart that is not found in the tricuspid and pulmonary valve endothelium. Induction of EphrinB2 expression promotes the remodeling action of the extracellular matrix which is mediated by increased smooth muscle levels induced by augmented actin production [19].

The augmented expression of EphrinB2 is one of the multiple mechanisms that promotes the adaptability of the leaflets of Pas when they are implanted in the aortic position. This characteristic offers a PA a way to accommodate the mechanical stresses of the new environment in which it is located, and it is mediated by reversible phenotypic changes allowing the acquisition of the morphofunctional characteristics typical of normal aortic valve leaflets [19]. The main effect is an increase in thickness and breaking point of the leaflet of the PA, which therefore takes on characteristics more similar to those of the valve leaflets of the native aorta in withstanding greater mechanical stresses [20].

Carr White et al. suggested that the use of PAs for aortic valve replacement was associated with survival benefits in patients who were randomized to receive an aortic valve replacement with an aortic homograft or a pulmonary autograft. Recipients had completed somatic growth and PAs were evaluated based on their mechanics and morphostructural profile [30]. The findings revealed significant structural and functional changes in the implanted PAs at the end of somatic growth as compared with the aorta of normal age-matched organ donors. The investigators observed that both in homografts and PAs, significant progressive dilatation of the aortic root did not occur. The expansion was defined as a dilative process of the aortic root at any level—annulus, sinotubular junction, and Valsalva sinuses—with a range > 20%. In addition, no more than mild aortic regurgitation was revealed in either group. However, in vitro histopathological analysis disclosed remarkable differences in the anatomic structure and mechanical features of pulmonary autografts. In particular, the tunica media of PAs tended to be thicker while the elastic fiber component of aortic homografts recorded minimal or no change. On the contrary, a degenerative process with considerable variation in the fragmentation of the elastic fiber architecture occurred in the pulmonary autografts. Importantly, the biomechanical behavior of PAs revealed a well-defined adaptation to pressure-mediated mechanical deformation, despite the differences in the stiffness modulus and maximum tensile strength in the explanted autologous tissue after 4 months. The investigators focused their attention on the proximal and distal suture line to explain the absence of progressive dilatation of the aortic root, showing that the variations in surgical technique, the orientation of the autograft, and the sizing match of the two vessels at the site of the anastomosis can influence the success of the Ross operation over time [30].

Chiarini et al. studied, by means of proteomic analysis, the dilated pulmonary autograft tunica media compared with normal pulmonary artery and aorta tissue. The investigators noted the upregulation of some proteins with specific functions in non-reinforced and dilated pulmonary autografts. Likewise, a downregulation was disclosed at all levels of genes coding for proteins related to focal adhesion (e.g., paxillin), cytoskeleton (e.g., vimentin), and metalloproteinase-regulating proteoglycans (e.g., testican-2). Microfibril-associated glycoprotein1, which controls elastic fiber accumulation, experienced a significant decrease. In addition, remarkable modifications of proteins deputed to the regulation of cellular signaling were reported, including an increase in the soluble Jagged-1 fragment, and the ectodisplasin-2 receptor associated with a decrease in the Notch-1 intracellular domain fragment. Furthermore, dilated non-reinforced pulmonary autografts revealed a substantial difference in Paxillin, Vimentin, the Jagged-1 fragment, and the Notch1 intracellular domain fragment as compared to those of control aortas, suggesting a maladaptive remodeling process that occurs in dilated non-reinforced PAs. The investigators obtained these results from non-reinforced pulmonary autografts, leaving the discussion open when comparing proteomic changes occurring in PAs reinforced with synthetic or biocompatible materials [96].

In our experimental studies, resorbable polyester supports were used, suggesting the potentiality of these materials to enhance the remodeling ability of pulmonary autografts [21,22,23,24,25,26,27]. The interaction between a bioresorbable reinforcement and PAs orchestrated an intricate vascular remodeling adjustment that was directed by a balance between inflammation and the production of an extracellular matrix (ECM). The result was the generation of a “neovessel” at the end of the biomaterial resorption phase. This newly organized structure experiences peculiar characteristics similar to those of the aorta, in that it is biologically alive and capable of growing. From a histological point of view, the ECM of reinforced pulmonary autografts revealed a greater amount of elastin fibers, as well as a more organized collagen fiber structure especially located in the elastic zone of the vessel. Of note is that metalloproteinase MMP-9 was overexpressed, thus explaining the ongoing remodeling process of the ECM. Likewise, cell proliferation was increased in association with a decrease in the apoptotic process, further supporting the evidence for active cellular remodeling and growth [22,23,26]. The use of a semi-resorbable reinforcement composed of resorbable polyester and an expanded polytetrafluoroethylene mesh offered promising results, especially in attenuating the effect of systemic loading pressure soon after implantation [21,22,23,24,25,26,27]. 

### 3.3. Hemodynamic Performance of Pulmonary Autograft

Regarding the haemodynamic performance, pulmonary autografts have shown a clinical benefit compared to the conventional prosthesis. In fact, mechanical and bioprosthetic valves secure the annulus, thus being intrinsically obstructive, while pulmonary autographs preserve the mobility of all the constituents of the aortic root. This feature promotes superior hemodynamic performance when PAs are used compared to that seen in a conventional aortic valve replacement (AVR). Um et al. reported that the use of PAs was associated with notably lower mean aortic gradients at discharge and follow-up as compared to the use of conventional mechanical and bioprosthetic valves for AVR [97]. These results are relevant because they demonstrate how small fluctuations in the trans-aortic gradient can have a clinical significance in reducing the risk of persistent cardiac insufficiency in individuals requiring valve replacements [98]. The effects of the use of PAs in improving hemodynamics are also due to the restoration of normal physiology, which leads to both an improved coronary flow reserve [99] and a greater regression of the ventricular mass [100,101]. However, computational models of biomechanics are not available to support this hypothesis.

A study performed with the use of magnetic resonance imaging and assessing flow patterns in various types of aortic root replacement procedures disclosed that the pattern and velocity of blood flow through pulmonary autografts were most alike to normal controls as compared to aortic homografts and bioprosthetic roots [102].

Although allogenic and autologous substitutes recorded similar hemodynamic performance with mean and peak transaortic gradients <10 mm Hg in the vast majority of individuals [97], pulmonary autografts revealed minimal calcification or degeneration, whereas many individuals develop high transaortic gradients following bioprosthesis or homograft implantation [103,104,105,106,107]. This implies that the transvalvular gradient remains stable over time with the use of the PAs as reported in long-term follow-ups, whereas it tends to increase after the implantation of homograft or bioprosthetic conduits [48,108,109,110,111]. The presence of low transvalvular gradients is even more significant when we consider the population of young individuals who want to exercise after AVR surgery. The hemodynamics of PAs are comparable with those of a normal aortic valve [99,100,101]. Further comparative studies based on computational models with the application of finite element analysis (FEA) and fluid dynamics tests may be useful to confirm the hemodynamic advantages of PAs compared to other valve substitutes.

## 4. Evidence from Deploying Mechanical Testing: Pulmonary Autograft Targets and Mechanisms of Action in Growing Tissue

Evidence has suggested that the progressive expansion of pulmonary autografts after the Ross procedure may reflect an inappropriate remodeling process involving the native pulmonary root, which must work to adapt its structure to the systemic circulation. A better understanding of the biomechanical mechanisms involved in autograft root dilation can offer valuable support for implementing strategies to prevent dilation. Although the normal human pulmonary root material properties have previously been characterized [112], the mechanical properties of failed autografts have only recently been investigated with the support of finite element analysis (FEA) [25,26,28,29,51,52].

### FE Simulations

Mookhoek et al. [51,52] and Nappi et al. [25,26,28,29] first independently reported the use of FEA methods to evaluate the mechanical performance of pulmonary autografts subjected to greater stress caused by systemic pressure.

Mookhoek et al. [51] studied the biomechanics of failed pulmonary autografts compared with normal pulmonary roots with the use of FEA simulation. The investigators worked on failed PA samples obtained from patients who had undergone reoperation after a previous Ross operation. The control group consisted of fresh human native pulmonary roots that were collected from the transplant donor network. The mechanical properties of the tissues were determined by performing biaxial stretch testing [113]. Instead, tissue stiffness was measured by patient-specific physiological stresses subjected to pulmonary pressure [51]. 

Most of the evidence derived from the study of Mookhoek are based on the following key points:

1. The constitutive modeling of the explanted PA and pulmonary roots were assumed to be incompressible and nonlinear hyperelastic materials,
2. Planar forces calculated by load cells during deformation were metamorphosed to Cauchy stresses in the principal longitudinal and transversal directions,
3. A nonlinear regression Levenberg–Marquardt least-squares algorithm in MATLAB (version 7.0.1, Natick, MA, USA) was used to adapt experimentally gained stresses to the corresponding theoretically measured stresses for explanted autograft and pulmonary roots.

The investigators revealed that a nonlinear stress–strain response was available in both the failed autografts and normal pulmonary roots. The explanted pulmonary autografts were less rigid when compared to their native pulmonary root counterparts at 8 mm Hg (*p* = 0.086) and 25 mm Hg (*p* = 0.006). Second, the stiffness of the autograft wall at both 8 and 25 mm Hg was not related to the relative age at which the Ross procedure was performed (*p* = 0.898 and *p* = 0.813, respectively) or with the time during which the PA was subjected to the highest pressure stress in the systemic circulation (*p* = 0.609 and *p* = 0.702, respectively). Finally, the failed pulmonary autografts retained a nonlinear response to mechanical loading typical of healthy human arterial tissue. These results suggested that the establishment of a remodeling process despite the increasing wall thickness nevertheless caused a reduction in the wall stiffness in the failed autografts. Therefore, the acquisition of greater compliance mediated by favorable remodeling offers a possible explanation for the progressive dilation of autograft roots in individuals who presented with autograft failures [51].

In a second report, Mookhoek et al. [52] compared the mechanical properties of explanted autografts to native aortic roots at systemic pressures. Autograft specimens were collected from patients who required reoperation due to PA failure after the Ross procedure. The investigators compared this group with native aortic roots that were obtained from unutilized donor hearts. The determination of the tissue mechanical properties was performed with the use of biaxial stretch testing. Instead, the tissue stiffness was measured at patient-specific physiologic stresses corresponding to systemic pressures at 80 and 120 mm Hg and when hypertensive conditions at 200 mm Hg were induced. Evidence revealed that nonlinear stress-strain curves were recorded for both failed pulmonary autografts and native aortic roots. The investigators highlighted the following findings: Firstly, the explanted autografts were markedly more compliant than native aortic roots at the following different systemic blood pressure measurements: 80 mm Hg (1.53 ± 0.68 vs. 2.99 ± 1.34 MPa; *p* = 0.011), 120 mm Hg (2.54 ± 1.18 vs. 4.93 ± 2.21 MPa; *p* = 0.013), and 200 mm Hg (4.79 ± 2.30 vs. 9.21 ± 4.16 MPa; *p* = 0.015). Secondly, the rigidity of the PA tissue measured at 80, 120, and 200 mm Hg of systemic pressure was not related to the age of the patient at the time of the insertion of the pulmonary autograft (*p* = 0.666, *p* = 0.639, and *p* = 0.616, respectively) or even at time of PA implant in the systemic circulation (*p* = 0.635, *p* = 0.637, and *p* = 0.647, respectively). The most-derived evidence disclosed that the failed pulmonary autografts preserved a nonlinear response to mechanical loading distinctive of healthy arterial tissue. Despite the similar wall thickness between autografts and aorta, autograft stiffness in this patient population was significantly decreased compared with native aortic roots. Mookhoek suggested that biomechanical remodeling was inadequate in specimens retrieved from patients who required reoperation. The PA did not reach the native aortic mechanical properties, which led to progressive autograft root dilatation. However, the authors did not clarify whether the patients were carriers of aortic insufficiency, which is a risk factor for the development of pulmonary autograft dilation [52].

**Figure 5:**
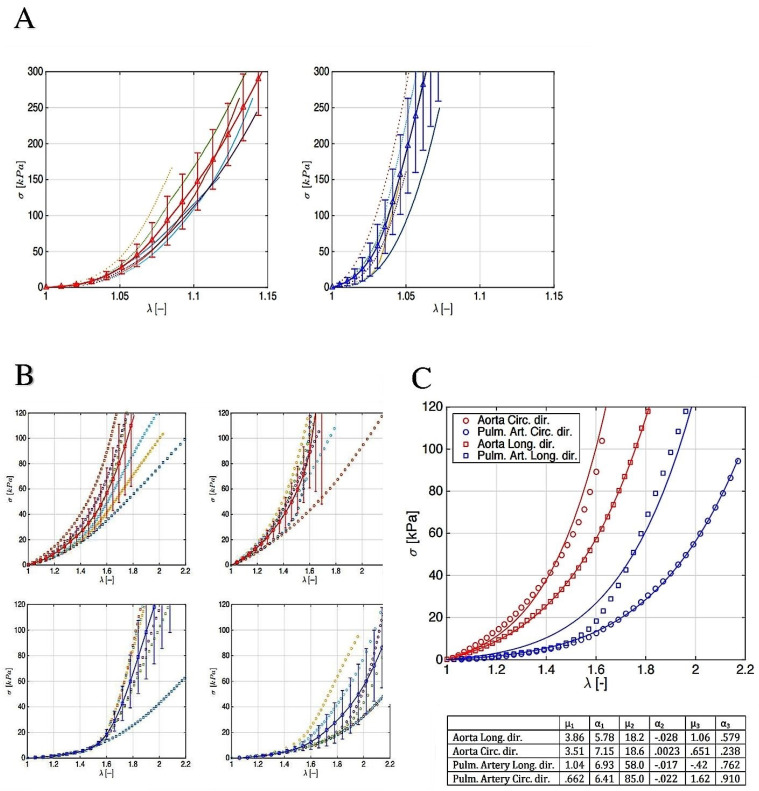
(**A**) Stress–stretch curves for pulmonary *(left*) and aorta (*right*) leaflet. (**A**) Pulmonary autograft (black arrow) and aorta explanted (red arrow) at 1 year. (**B**) Stress–stretch curves for aorta: longitudinal (*top–left*) and circumferential (*top–right*) direction; stress–stretch curves for pulmonary artery: longitudinal (*bottom–left*) and circumferential (*bottom–right*) direction. (**C**) Synoptic of the (*top*) average stress–stretch curves for both aorta and pulmonary artery along with the two mechanically relevant directions (see legend for details) and (*bottom*) a table with the fitting parameters.

**Figure 6:**
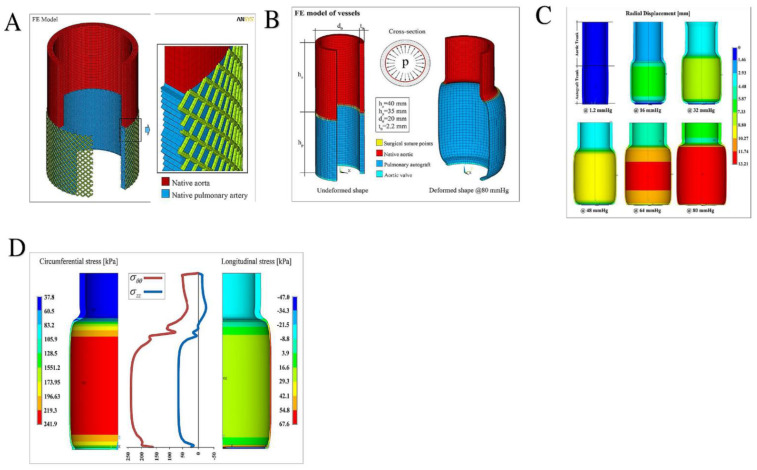
(**A**) Left: pulmonary autograft reinforced with semiresorbable prosthetics integrated in pulmonary autograft wall. Right: geometry of the FE model, with a detailed correlation of the native aorta (in red) and the pulmonary artery tract (blue), integrated with the external e-PTFE structure at the time of full development (**B**) Overall sketch of the Finite Element model reconstruction of the aorta-suture-autograft-annulus ensemble: undeformed system (left); deformed (at the maximum pressure level) model (right) and cross-section with applied pressure. At the bottom, the legend with the details of the elements used, distinguished for material properties. (**C**) Sequence of deformations at increasing pressure levels up to 80 mm Hg. The contour plots refer to the displacements along the radial direction (in mm). (**D**) Hoop (circumferential) and longitudinal (axial) stress profiles as a function of the vessel axis (middle), with contour plot details showing the spatially inhomogeneous distribution of the stresses (in kPa). Abbreviations; e-PTFE; expanded polytetrafluoroethylene.

We described the uni-axial tests along with the mechanically pertinent directions, i.e., the longitudinal and circumferential ones, that were calculated for the aorta and PA roots. Given the stress–strain outlines, it emerged that the hyperelastic responses of PAs and aorta roots were anisotropic, with a classical increasing slope as the stretch grew. The aortas displayed a stiffer behavior in both the hoop and axial directions compared to the PAs, thereby confirming the most relevant reports in the literature. In addition, the strength values appeared to corroborate the mechanical resistance hierarchy, with the stress threshold being higher in the aorta with respect to the PA equivalent. Biomechanically substantial evidence suggested that the stress–strain response of the aorta and PA valve leaflets had very similar qualitative and quantitative behaviors, which were evinced by both tissues (aortic wall and leaflet structure) up to the applied forces and prescribed stretches. Two independent reports reached the same conclusions, demonstrating a high degree of mechanical strength and durability of the pulmonary valve when it was transposed to the aortic position in a Ross operation, despite PAs being subjected to high-pressure regimes [23,28,53] (Figure 5 and Figure 6).

For the analyses, the pressure values increasing within the physiological range up to 80 mm Hg were considered, corresponding to a maximum (circumferential) stress in the PA of about 240 kPa and an expansion of the diameter greater than double the undeformed one (panel A and B), coherently with the experimental stress–strain measurements both in the reinforced (panel A) and in the non-reinforced PA (panel B–D). In particular, panel C and D showed that the simulation outcomes were synoptically reported. Again, in panel C the sequence of the overall deformation of the system with increasing applied pressure was collected. 

Evidence from the contour plots disclosed that the radial displacement nonlinearly increased with the exerted pressures, generating significant strain gradients along the longitudinal direction (i.e., the vessel axis), which can be traced as the primary culprit for aneurysms (panel C). It has to be noticed that the bulging shape of the deformed autograft, induced by both the discrepancy in stiffness between aorta and PA and the constraint of the basal annulus, determined the radial displacement gradients associated with migration of the suture section upwards as a result of the competition with the adjacent aorta. In addition, we recorded that the measurements conducted at a pressure higher than about 80 mm Hg would kindle exceedingly localized strains and instability phenomena at the level of the suture regions. This evidence was in agreement with the expected inelastic (irreversible) deformation processes preceding tissue damage and failure in the absence of any PA reinforcement (panel A and panel D). In fact, in panel D, the corresponding hoop stress distribution, at the maximum pressure level, revealed a strong variation along the vessel axis (z-direction), with localized stress gradients at the PA–aorta connection at the level of the region corresponding to the suture. In addition, as a consequence of the advised boundary conditions, the longitudinal stresses disclosed a change in sign along the vessel axis, passing from tensile regimes in the PA to low compressive values in the aorta tract [25,28,29]. 

## 5. Remodeling Induced with the Use of Polyester: Crosstalk between Biophysical Features and Clinical Prosthetic Use

Polydioxanone (PDS; Ethicon Inc. Johnson & Johnson, Bridgewater, NJ, USA) is a polyester with hydrogel-like characteristics. It can be reabsorbed within biological systems undergoing progressive degradation without this leading to nuanced reactions or toxic effects. These peculiar characteristics have been exploited in our experimental studies to reinforce the pulmonary artery wall [22,23,24,114]. The degradation and elimination of polydioxanone are determined by the process of cell phagocytosis, enzymatic degradation, and physical dissolution through biological liquids. PDS has been used as an external resorbable reinforcement prosthesis and has proved to be versatile and not change its features during the initial time. During the period in which the material was inside the vessel wall, from 6 months to 12 months later, the implantation in growing lambs did not evoke any inflammatory reaction requiring its removal. In addition, no cases of blood clots were recorded. After complete resorption of the initial implant polyester in the extracellular matrix (ECM), no detrimental histological reactions were noted but a physiological remodeling process in the ECM of the vessel wall was reported [21,22,23,24,26,27,114,115,116,117].

PDS has been shown to be a biomaterial capable of interacting with the elastic properties of the pulmonary artery. Furthermore, a synergy was found between the elastic properties of the PDS and the degradation time that was required once implanted, obviously in accordance with the application for which it was intended. It is an ideal polymer with the biological and mechanical properties outlined in [22,26,27,55,114,116]. As for the biological properties, firstly, the PDS, once in contact with the morphological structure of the pulmonary artery, did not evoke excessive toxic or inflammatory responses of the ECM. Secondly, it was metabolized, after having completed its favorable strengthening and remodeling action, without leaving a trace. Thirdly, the PDS was easily processable [21,22,23,24].

The mechanical properties of PDS were evaluated according to the application of the material, which was to contain the expansion of the pulmonary artery when it was implanted under systemic pressure, so that the polyester load was automatically distributed on the pulmonary artery tissue as it was degraded, thus reducing the effect of stress shielding [23,24,26].

### 5.1. Specific Characteristics of Polydioxanone

Polydioxanone (PDS) has about 55% crystallinity and a glass transition temperature that varies between −10 °C and 0 °C. It features a regular repetition of fundamental units that come together in chains folded into dense regions called crystallites. These join together by means of cross-links, giving the polymer a high tensile strength and a very high elastic modulus if compared to that of the amorphous analog. These features are related to the degree of compactness of the polymer. It should be noted that no polymer can be organized into a total crystalline structure, so however high a crystallinity may be found inside, partially amorphous regions will always be detectable. PDS is a very viscous, high–medium molecular weight polymer that has slower biodegradation than those with a lower molecular weight and lower viscosity. Notably, temperature plays a fundamental role with regards to its material properties [21,22,23,24,26]. 

### 5.2. Biodegradation Molecular Mechanisms

PDS is degraded primarily due to the loss of molecular weight and the loss of resistance of the material through an initial phase in which the degradation is due to a process of a chemical nature. The biological process and the total removal of the material takes place later [22,23,24,114].

In detail, polydioxanone polymers are degraded in two ways: dissolution and chain cleavage. In the case of dissolution, since PDS has hydrophilic domains, it is dissolved during normal physiological processes when solvent molecules, such as H_2_O present in pulmonary artery tissue, are absorbed into the polymer and are small enough to occupy the space between the chains of the macromolecules. The H_2_O molecules that penetrate into the polymer act as plasticizers, thus making the material more ductile as they reduce the number of secondary bonds between the chains. Furthermore, it is possible that this process could cause an altered crystallinity of the polymer. Both mechanical and thermal properties (e.g., glass transition temperature) can be affected by the absorption of solvent molecules. It is also possible that, in extreme cases, the chains are soluble enough that few covalent bonds remain between the chains and the polymer that dissolves completely [22,23,24,114].

Instead, the chain splitting involved the breaking of the primary bonds, rather than the secondary ones. There is a separation between the chain segments at the breaking point of the bond, which leads to a reduction in the molecular weight, which can have, as in the case of dissolution, significant consequences on the mechanical and thermal properties. Chain splitting can occur hydrolytically or by oxidation [21,22,23,24,25,26,95,114]. 

Regarding hydrolysis, the H_2_O molecules penetrate the implanted material causing the splitting of the molecular bonds between the monomers. This condition leads to the splitting of the polymer chains into shorter chains. The main factors that produce the extension of PDS hydrolysis are: the reactivity of the functional groups of the main part of the polymer; the extension of the inter-chain links; and the increase in the H2O available for the polymer. We have recorded non-degraded PDS in the vessel wall segments due to probable reduction in the tissue H_2_O content, but we cannot establish whether it is secondary to dissolution or hydrolytic cleavage [21,22,23,24,25,26,95,114].

In the pulmonary artery wall, PDS is also eliminated by oxidation through the formation of free radicals that attack and break the covalent bonds that hold the chains together. The main factors influencing oxidation are mostly the reactivity of the functional groups of the main part of the polymer and the extension of the inter-chain links [21,22,23,24,25,26,95,114]. 

The degradation of polydioxanone occurs through the cleavage of their ester chains. PDS, on the other hand, is split into glycoxylate and excreted in the urine, or converted into glycine and subsequently, similarly to Polyglactin (PGA), transformed into H_2_O and carbon dioxide. In any case, both PGA and PDS follow the same final cycle (from pyruvate onwards) as PLA. The degradation time, as already mentioned, depends on the porosity of the copolymer, crystallinity, and molecular weight. Polyglactin and PDS materials can be selected as early- and late-resorbable materials, respectively, with an absorption time of ~21 days for PG, and ~6 months for PDS. In addition, PDS is used as the preferred suture material in pediatric cardiac surgery [21,22,23,24,25,26]. 

### 5.3. The Use of Polydioxanone as Crosslinked Prosthetics: When and How

The pulmonary valve and trunk or a pulmonary autograft (PA) may be used to treat aortic valve disease, however, the pulmonary conduit is exposed to progressive expansion when it is implanted under the systemic pressure regimen. This operation was firstly performed by Donald Ross in 1967 and was hence eponymously coined [1] (Figure 1 and Figure 2).

The checks performed by our clinical teams confirmed a trend towards the dilatation of pulmonary autografts after Ross procedures leading to a potential risk of reoperation. The use of Dacron as an external reinforcement is a choice adopted by many surgeons, however, we never were in favor of adopting it. We preferred to maintain the characteristics of PAs as living tissue while trying to induce the remodeling of the pulmonary conduit [6,7,28,53,54,106]. 

Dacron, for various reasons, cannot be considered a suitable material for this reinforcement and the late outcomes of the use of these synthetic materials are increasingly revealing its limitations at both the clinical and biological levels. In fact, the Dacron used by many surgeons in many institutions is unable to grow and fails to match the demanding needs of a growing structure such as pulmonary autografts, especially when it is implanted in the pediatric population [29,103]. Secondly, its vascular compliance is poor, and it has been estimated that Dacron has a stiffness that is 24 times greater than the native aorta [21,22,23,24,114]. This would imply the loss of elastomechanical properties and the Windkessel function of the neoaortic root with a retrograde effect on the aortic valve, crowding its leaflet and eventually leading to its incompetence. Lastly, synthetic materials can induce a strong inflammatory reaction impairing PA graft viability and interfering with the arterialization process. This concept has been recently stressed in the literature, which is increasingly pointing at the graft viability and its biological features as one of the main reasons for the clinical success of the Ross operation [25,29,115].

**Figure 7:**
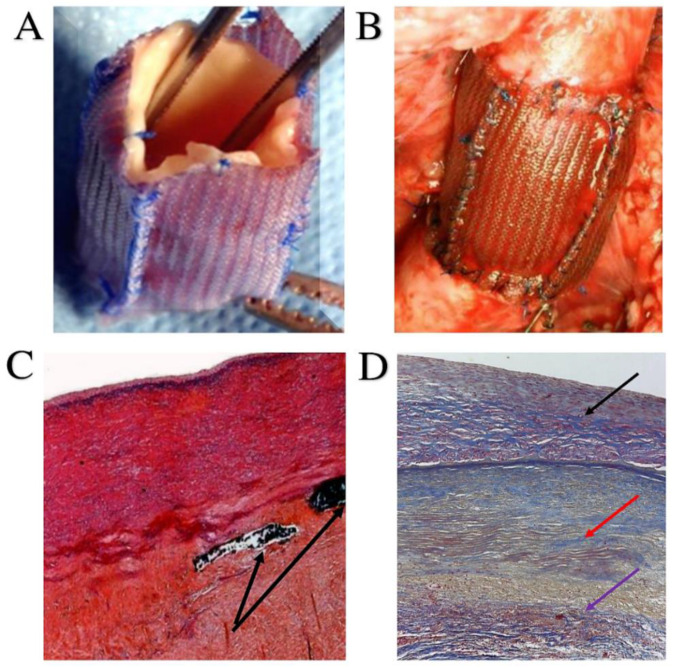
(**A**) Pulmonary autograft reinforced with 15 mm-wide bands of knitted PDS. (**B**) PA implanted in aortic position. (**C**) The media of pulmonary autograft is perfectly intact. Remnants of the slowly resorbable material of PDS are highlighted in the adventitia (black arrow). (**D**) The histology of PA revealed a complete resorption of the PDS mesh with no damage to the media and an increase in the regenerative connective component especially at adventitial level. This regenerative tissue was found to be constituted by elastic fibers as can be seen in the Masson’s Trichrome staining. Black arrow shows the tunica media with normal thickness and no disruption, red arrow reveals elastin fibers and violet arrow highlights no inflammatory reaction. Abbreviations: PDS, polydioxanone (Ethicon Inc. Johnson & Johnson; Bridgewater, NJ, USA); PA, pulmonary autograft.

From a histopathological point of view, the use of external Dacron reinforcement is accompanied by remarkable thinning of the pulmonary artery wall leading to intimal denudation and multiple medial disruptions. Dacron meshes can migrate inside the vessel wall. We recorded a high percentage of disseminated collagen fibers associated with peculiar histological alterations that showed a conspicuous inflammatory infiltrate [21,22,23,24,26,114,115,116,117] (Figure 7A–D). 

On the contrary, pathological analysis performed after the use of resorbable polydioxanone to reinforce the pulmonary artery revealed partial resorption of the polyester, recording a preserved endothelial lining. This evidence confirms the absence of intimal tearing under pressure load. In addition, a medial thickening with thoroughly arranged fibromuscular cells mixed with abundant neo-formed connective tissue was identified. Notably, using the polydioxanone, MMP-9 was detected to be overexpressed once the polyester was integrated into the ECM, pointing out an ongoing matrix remodeling process [22,24,26,27,114,116]. The collagen architecture appeared well organized with a compact density and distribution in the elastic region of the pulmonary artery. Taken together, these results concretely suggest a shift towards a process of elastic remodeling and neo-arterialization. In confirmation of these findings, it was possible to note the presence of scarce inflammatory infiltrates with rare macrophages or monocytes that colonized the reinforced arterial wall. Pathoanatomical evidence reliably suggested that the biomaterial did not elicit an exuberant foreign body inflammatory reaction over time [21,22,23,24,26,116,117]. 

We induced neoarterialization of the PA, which was placed in the aortic position and therefore exposed to systemic pressure. In the pulmonary artery, we reproduced the physiological condition according to which, under the effect of systemic pressure, a conversion of the elastic tension into elastic potential energy occurred. The previously reported histological evidence demonstrated that during the reabsorption process of a scaffold constituted by PDS and applied to the PA, a remodeling phenomenon of the vessel wall occurred, resulting in a denser connective architecture of the tunica media with an increase in its elastic component [21,22,23,24,27] (Figure 7C,D). 

**Figure 8:**
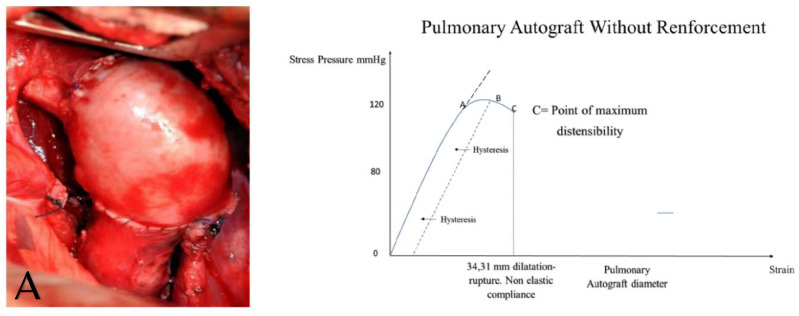

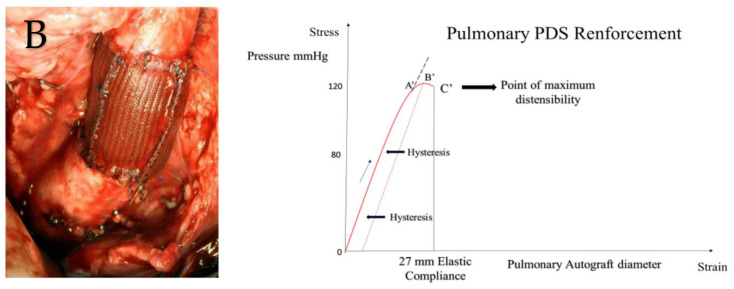
The ability of resorbable hydrogels such as PDF to modify the elastomechanical function of the vessel wall. The application of a bioresorbable reinforcement with the function of hydrogel is able to modify the behavior of the curve of distensible materials as the PA wall, obtaining an increase in their elastic properties. This is observed in the curve as the shift of A (**panel A** left) toward A′ (**panel B** left) with increased elasticity and compliance. The maximum distensibility, point C toward C′, as the reinforcement effectively prevented massive dilation. This provoked a reduction in the critical area determined by the fall of the curve (**panel B** left). The potential elastic energy depended on the extension of the surface and on the composition of the material constituting the cylinder including the intramolecular cohesion forces. We demonstrated histologically that during the resorption process of a PDS scaffold applied to the PA (**panel B**), a remodeling process of the vessel wall occurred, resulting in a denser connective architecture of the tunica media with an increase in its elastic component.

The bioresorbable polyester external prosthesis increased the surface of the neoaorta and, at the same time, was reabsorbed within the PA in order to modify both the cohesion forces and the elastic characteristics of the membrane. This process was possible for the molecular structure of PDS, which is characterized by the repetition of fundamental units, with a crystallinity of about 55%, capable of increasing the intramolecular cohesion forces [7,21,22,23,24,106,114] (Figure 8A,B). 

## 6. Biomechanics of Pulmonary Autograft Leaflet and Root: Clinical Application

Several studies evaluated the biomechanical properties of leaflets and the arterial root of PAs and native aortas with regards to monoaxial and biaxial circumferential and longitudinal stresses, to whom pulmonary autografts were exposed after transposition in the aortic position and increased pressure stress [28,30,51,52]. 

Our evidence confirmed the previous study of Carr-White et al. who evaluated the mechanical behavior of PAs after a monoaxial stress test [30] and increased the results published by Horer et al. [35] offering a definitive response with regard to the non-linear behavior between the growth, remodeling, and stress shielding of pulmonary autografts subjected to a high-pressure regime.

We learned that PA valve regurgitation occurs at a rate of 40% of individuals 20 years after Ross operations despite the pulmonary valve being free from dysfunction at a rate of 53.5% of individuals [50]. Likewise, evidence from a large clinical series does not definitively indicate the preferred treatment strategy to prevent the complication of PA expansion. Although many observational studies suggest the benefits of adding external reinforcement to PAs [13,28,34,37,47,67,78,118,119], there are others that revealed negative or neutral findings [13,30]. 

We worked on the importance of the biomechanical features of reinforced and non-reinforced PAs [25,28,29], extending the evidence originally published by Horer [35] and subsequently reported by Mookhoek et al. [51,52]. The latter, while confirming the nonlinear response to mechanical load in failed pulmonary autografts, on the other hand recorded increased compliance and reduced wall stiffness of PAs when compared to native pulmonary arteries, suggesting an explanation for PA dilation [52]. In addition, Mookhoek et al., when comparing failed PA roots to native aortas, disclosed an increased compliance of explanted PA roots with respect to the aorta; however, PAs revealed inadequate biomechanical remodeling, which did not match the characteristic wall stiffness of native aortas [51]. 

Our findings indicated the importance of the remodeling phenomena occurring in PAs and the reflexes on the biomechanical properties of remodeled autografts by means of an in vivo model of Ross operations. We also described the stress shielding effects that a resorbable mesh might exert when it is used as a reinforcement for a PA [21,22,23,24,25,26,28]. Again, we previously revealed the detrimental effect of Dacron grafts and other synthetic polyesters that gravely impair aortic compliance when used as external pulmonary artery reinforcements, thereby triggering a strong inflammatory reaction with significant injury to the vessel wall and negative stress shielding effect on the distal suture [29,115]. 

The results of the FEA suggested a close interaction between the material properties of autografts and aortas, the suture regions, the geometry, and the dilation constraints imposed by the annulus. This orchestration plays a crucial role in determining the effects that actual stress concentrations, initiation of strain localization, and strain gradients have on the success of Ross operations [28,29]. 

In clinical applications, it is important to emphasize that while the aorta recorded a consensual rise in stress and deformation in both directions, pulmonary autografts revealed better adaptability in the longitudinal direction and a steeper curve in the circumferential response. This evidence suggested the following certainties: Firstly, the ability of PAs to evenly absorb mechanical stresses and potentially explain their known dilatation tendency over time. Secondly, a greater degree of resistance to deformation of valve leaflets with a stiffer behavior in respect to the aorta for applied loads of about 240kPa (1800 mm Hg) was demonstrated [6,7,8,20,28,30,101]. 

In parallel to what was reported by Mookhoek et al., the significant value of the physiological pressure considered for the analysis was 80 mm Hg. Furthermore, the FE analyses were also carried out in a static regime, thus neglecting the effects of inertia, and standard convergence algorithms were also used to follow the non-linear procedure relating to the presence of both large deformations and hyperelastic behavior. Likewise, the results for the different thicknesses (2–2.5 mm) revealed that circumferential stress peaks were found in the range 118–158 kPa for aortic roots and 206–277 kPa for native PAs, consistent with those obtained in the simulations performed by Mookhoek et al. A similar comparison can be made in terms of thicknesses and deformed diameters, where the greatest discrepancies for PAs were recorded [51,52].

Evidence on the biomechanics of PAs supports a reconsideration of the assumption of a linear relation in the dimensional expansion of PA structures even more with regard to the age of the implant of the pulmonary autograft and providing crucial importance to the phase of somatic growth. In this context, our findings corroborate the observations previously described by Horer et al. [6,7,15,28,35,61,62,84,85,86,87,88,89,90,91,95] as to the increase in the diameter of the annulus, Valsalva sinus, and sinotubular junction of the PA year on year. [28,35] Again, the length of the pulmonary autograft inserted as well as the technique of implantation requires specific tailoring to reach good long-term follow-up [6,7,30,48]. With the exception of the annulus, which proved to be less deformable than the PA root, it instead recorded a consensual increase in the longitudinal and circumferential stresses using a significant load. These findings offer an explanation supporting the stress shielding feature of the PA root, which allows for uniform distribution of forces within the walls and could provide a relatively long life for the PA [6,7,28,30,35,48,51,52].

## 7. Limitations

This study has several limitations. It is a systematic review of the best available evidence which itself has the inherent risks of pooling data from multiple studies which are heterogeneous. Given the specialist nature of the subject, selection bias may have affected the observed outcomes with the high-volume centers preferred. In addition, an assessment of bias and a metanalysis were not conducted.

## 8. Conclusions

The Ross procedure has excellent durability and longevity in clinical and biomechanical studies. The use of external reinforcements such as semi-resorbable scaffolds may further extend the lifespan of these valve substitutes. The inherent adaptability of PAs accounts for some of their excellent outcomes alongside surgical techniques and tissue handling. 

## Figures and Tables

**Table 1:** Overview of studies obtained by systematic review reporting young adult and pediatric series of Ross operations.

First Author/Year of Publication (Ref.)	Study Type	Period of Surgery	Number of Patients (N)	Mean Age, y (Range)	Surgical Technique	Biomechanical Findings
Aboud 2021 [61] JACC *Germany*	Retrospective	1988–2001	2244	33 (16–61)	RR Root SC	† At 25 yrs excellent biomechanical functioning in unreinforced PA root with slight dilatation. No dilatation in RR. Excellent biomechanical functioning of PA valve in SC implantation
Nappi 2018 [28] *ICVTS* *France*	Retrospective	1998–2002	66	29 (16 mth–62)	RR Root SC	† At 22 yrs excellent biomechanical functioning in unreinforced PA root with slight dilatation. No dilatation in reinforced Ross. Excellent biomechanical functioning of PA valve in SC implantation
Sievers 2016 [37] *EJCTS* *Germany*	Retrospective/Prospective	1990–2013	1779 569 (16–40 yrs)	31 (16–40)	RR Root SC	† At 20 yrs Excellent biomechanical functioning in unreinforced PA root with slight dilatation. No dilatation in reinforced Ross. Excellent biomechanical functioning of PA valve in SC implantation
Andreas 2014 [47] *Annals* *Germany*	Retrospective	1991–2011	246	25 (5–46)	RR Root	Slight dilatation in PA root implanted at STJ with excellent biomechanical performance
Da Costa 2014 [46] *Eur J Cardiothorac Surg* *Brasil*	Retrospective	1995–2013	441	31 (5–56)	Root/IC/ SC	Slight dilatation in PA root implanted at STJ with excellent biomechanical performance. Excellent biomechanical performance of PA valve in SC implantation
Ruzmetov 2012 [62] *Ann Thorac Surg*. *USA*	Retrospective	1990–2011	106	18 (1 mth–40)	Root	Slight dilatation in PA root implanted at STJ. Biomechanical performance of PA root guaranteed no failure
Bohm 2009 [63] *Ann Thorac Surg.* *Germany*	Retrospective	1995-2006	467	41 (26–56)	Root/ SC	Slight dilatation in PA root with excellent biomechanical performance. No failure of PA valve with biomechanical performance in young adults with SC implantation
Elkins 2008 [13] *JTCVS* *USA*	Retrospective	1986-2002	487	24 (2–62)	Root SC	Slight dilatation in PA root reinforced at level of STJ. Excellent biomechanical performance of PA root and valve
Klieverik 2008 [64] *EHJ* *Holland*	Prospective	1987-2007	63	29 (16–52)	Root/IC	Excellent biomechanical functioning in reinforced PA root with IC procedure Slight dilatation of PA
Klieverik 2007 [65] *EHJ* *Holland*	Prospective	1988–2005	146	22 (0.3–52)	Root/IC	Excellent biomechanical functioning in reinforced PA root with IC procedure Slight dilatation of PA
Chiappini 2007 [66] *Ann Thorac Surg*	Retrospective	1991–2005	219	36 (0.5–64)	Root/IC/ Subcoronary	PA root reinforced with IC technique guaranteed slight dilatation and excellent biomechanical functioning
Pasquali 2007 [67] *JTCVS*	Retrospective	1995–2004	121	8.2 (0–34)	RK Root	No PA root dilatation in RR. No histological studies have tested the detrimental effect of Dacron graft on biomechanics of RR
Brown 2007 [34] *Ann Thorac Surg* *USA*	Retrospective	1993–2005	170	25 (0–61)	RK Root	No PA root dilatation No histological studies have tested the detrimental effect of Dacron graft on biomechanics in RR
Kumar 2005 [68] *Ann Thorac Surg*	Retrospective	1993–2003	153	28 (0–65)	Root	Optimal biomechanical performance in PA root with slight dilatation. No use of external reinforcement
Kumar 2006 [69] *Eur J Cardiothorac Surg.*	Retrospective	1993–2003	81	21 (0–51)	Root	Excellent biomechanical functioning in PA root with slight dilatation. No use of external reinforcement
Kouchoukos 2004 [70] *Ann Thorac Surg* *USA*	Retrospective	1989–2002	119	31 (5–56)	Root	Optimal biomechanical functioning in PA root with slight dilatation. No use of external reinforcement in RR
Luciani 2012 [71] *EJCTS* *Italy*	Retrospective	1994–2004	112	29 (6–49)	Root/IC/ SC	Slight dilatation in PA root implanted in IC. Biomechanical performance of PA root guaranteed no failure. Excellent biomechanical performance of PA valve in SC implantation and in IC technique
Raja 2004 [72] *BMC Cardiovasc Disord* *UK*	Retrospective	1996–2003	38	13 (1–30)	Root	Optimal biomechanical performance in PA root with no dilatation. No use of external reinforcement in RR
Alphonso 2004 [73] *Eur J Cardiothorac Surg.*	Retrospective	1991–2002	60	15 (0.5–67)	SC/IC	Very good biomechanical performance of PA valve in SC implantation using IC technique.
Sakaguchi 2003 [74] *J Heart Valve Dis*	Retrospective	1986–2000	399	23 (0–59)	Root/IC/ SC	Optimal biomechanical performance of PA valve in SC implantation using IC technique. Slight expansion in PA root implant
Concha 2003 [75] *Eur J Cardiothorac Surg*	Prospective	1991–2002	169	30 (0–54)	Root	Excellent biomechanical performance in PA root implant with slight expansion. No use of external reinforcement
Takkenberg 2002 [76] *Eur J Cardiothorac Surg* *Holland*.	Retrospective	1988–2000	343	26 (0–58)	Root/IC/ SC	Excellent biomechanical performance in unreinforced PA root using the IC technique. Slight dilatation. Excellent biomechanical functioning of PA valve in SC implantation
Pessotto 2001 [77] *Ann Thorac Surg.*	Retrospective	1992–1999	111	16 (0–67)	Root SC	No PA root expansion with optimal biomechanical performance in unreinforced root. No PA valve failure in SC implantation with excellent biomechanical functioning.
Laudito 2001 [78] *JTCVS*	Retrospective	1993–2000	72	9 (0–40)	RK Root	Preserved biomechanical features of PA root
Sharoni 2000 [79] *Isr Med Assoc J*. *Israel*	Retrospective	1996–1999	40	8 (0–41)	Root	Slight expansion of unreinforced PA root with preserved biomechanical features of PA root
Moidl 2000 [80] *J Heart Valve Dis.*	Prospective	1991	109	32 (6–59)	Root Subcoronary	Slight expansion of unreinforced PA root with preserved biomechanical features. Optimal performance of PA valve in SC implantation
Chambers1997 [31] *Circulation* *UK*	Retrospective	1967–1984	131	32 (11–52)	Root/SC	Slight expansion of unreinforced PA root with preserved biomechanical features. Optimal performance of PA valve in SC implantation
Matsuki 1988 [81] *JTCVS* *Japan*	Retrospective	1967–1986	241	(9–60)	SC	25 yrs follow up optimal performance of PA valve in SC implantation without failure
Gula 1979 [82] *Ann Thorac Surg* *Japan*	Retrospective	1967–1977	188	30 (9–64)	SC	Optimal performance of PA valve in SC implantation without failure
Somerville 1979 [83] *Br Heart J* *UK*	Retrospective	1967–1972	85	30 (12–54)	SC	Optimal performance of PA valve in SC implantation without failure

Abbreviations; IC, inclusion cylinder; PA, pulmonary autograft; RK; Ross–Konno; RR, root-reinforced; SC, subcoronary; † maximum follow up.

**Table 2:** Overview of studies obtained by systematic review reporting pediatric series of Ross operation.

First Author/Year of Publication/Location (Ref.)	Study Type	Period of Surgery	Number of Patients (N)	Mean Age, y (Range)	Surgical Technique	Biomechanical Findings
Stewart 2007 [84] *Ann Thorac Surg.*	Retrospective	1994–2005	46	13 (1–21)	Root	Optimal biomechanics with slight dilatation in PA unreinforced root
Ruzmetov 2012 [62] *Int J Cardiol* *USA*	Retrospective	1993-2005	81	<18 yrs	Root/IC	No dilatation in PA root with IC. Optimal biomechanical performance without failure in PA valve and root
Kalavrouziotis 2006 [85] *Hellenic J Cardiol.* *Greece*	Retrospective	1996–2004	35	10 (0.3–18)	Root	Optimal biomechanics of PA valve and root with slight dilatation of PA root
Bohm 2006 [86] *Ann Thorac Surg*. *Germany*	Retrospective	1995–2004	60	12 (1–20)	Root	Slight dilatation in PA unreinforced root. Preserved biomechanics of PA valve and root
Takkenberg 2005 [87] *Ann Thorac Surg.* *Holland*	Prospective	1988–2003	47	8 (0–15)	Root	Optimal biomechanics of PA valve and root in absence of IA. No dilatation in PA unreinforced root
Khwaja 2005 [88] *Semin Thorac Cardiovasc Surg* *Pediatr Card Surg Annu.* *USA*	Retrospective	1992–2005	53	14 (10–21)	Root	Slight dilatation in PA unreinforced root. Preserved biomechanics of PA valve and root
Hazekamp 2005 [89] *J Cardiothorac Surg.*	Retrospective	1994–2003	53	9 (0–18)	Root	Slight dilatation in PA unreinforced root. Optimal biomechanics of PA valve and root
Hraska 2004 [90] *Eur J Cardiothorac Surg*	Retrospective	1997–2003	66	13 (0–23)	Root/RK	No dilatation in PA-RR with Dacron graft. Very good biomechanical performance without failure in PA valve and root
Al-Halees 2002 [91] *J Thorac Cardiovasc Surg*	Retrospective	1990–2000	53	8 (0–18)	Root/IC	No dilatation in PA root with IC. Optimal biomechanical performance without failure in PA valve and root
Elkins 2001 (61) *J Heart Valve Dis* *USA*	Retrospective	1986–2001	178	10 (0–18)	Root/IC	No dilatation in PA root with IC. Optimal biomechanical performance without failure in PA valve and root

Abbreviations; IC, inclusion cylinder; PA, pulmonary autograft; RK; Ross–Konno; RR, root-reinforced.

## Data Availability

Not applicable.

## Supplementary Materials

The following supporting information can be downloaded at: https://www.mdpi.com/article/10.3390/bioengineering9090456/s1. Table S1: Prisma checklist for Biomechanics of Pulmonary Autograft as Living Tissue: A Systematic Review.

## Abbreviations

ACC/AHA	American College of Cardiology/American Heart Association
ESC	European Society of Cardiology
FEA	finite element analysis
IC	inclusion cylinder
kPa	kilopascal
PA	pulmonary autocraft
PDF	polidioxanone
RK	Ross-Konno
RR	root reiforgement
SC	subcoronary
